# Treatment outcomes of retreated patients with isoniazid/rifampicin resistant pulmonary tuberculosis

**DOI:** 10.1186/s12879-023-08909-2

**Published:** 2024-01-02

**Authors:** Lijie Zhang, Xiqin Han, Qiping Ge, Wei Shu, Yuxian Sun, Jingtao Gao, Shiheng Xie, Jingping Wang, Weiwei Gao

**Affiliations:** 1 Clinical Center on Tuberculosis, Beijing Chest Hospital, Capital Medical University/ Beijing Tuberculosis and Thoracic Tumor Research Institute, No. 9, Beiguan, Tongzhou District, Beijing, 101149 P. R. China; 2 Department of Tuberculosis, Beijing Chest Hospital, Capital Medical University/Beijing Tuberculosis and Thoracic Tumor Research Institute, Beijing, 101149 P.R. China

**Keywords:** Pulmonary tuberculosis, Retreated tuberculosis, Isoniazid resistance, Rifampicin resistance, Treatment outcome

## Abstract

**Background:**

About 8% of TB cases worldwide are estimated to have rifampicin-susceptible, isoniazid-resistant tuberculosis (Hr-TB), ranging from 5 to 11% regions. However, Hr-TB has not received much attention while comparing to be given high priority to the management of rifampicin-resistant tuberculosis (RR-TB). This study aimed to compare the differences of treatment effects for Hr-TB and RR-TB, so as to intensify the treatment and management of Hr-TB.

**Methods:**

A retrospective study was used to collect bacteriologically positive retreated patients with isoniazid/rifampicin resistant pulmonary tuberculosis, who were conducted at 29 tuberculosis control institutions in China from July 2009 to June 2021. We assessed effectiveness and safety of retreated patients with isoniazid/ rifampicin resistant pulmonary tuberculosis.

**Results:**

A total of 147 with either positive smear or cultures were enrolled, and 80 cases were in Hr-TB group and 67 cases were in RR-TB group. There was no significant difference in terms of age, sex, body mass, type of retreatment and comorbid diabetes between the two groups (*P* > 0.05). The rate of number of lesions involving lung fields ≥ 3 in Hr-TB group 75.9% (60/79) was significantly higher than RR-TB group 56.7% (38/67) (χ^2^ = 6.077, *P* = 0.014). There was no statistically significant difference (*P =* 0.166) with regard to the treatment outcomes of the two groups, the cure rates were 54.7% (41/75) and 53.6% (30/56), respectively, and the failure rate in Hr-TB group 22.7% (17/75) was 10% higher than RR-TB group 10.7% (6/56). The rate of negative sputum smear at the end of the second month (65.7%) in the Hr-TB group was significantly lower than that in the RR-TB group (85.7%) (*P =* 0.025). There were no significant differences in the incidences of serious adverse reactions and chest X-ray changes between the two groups (*P* > 0.05). During the 5-year follow-up, recurrence in the Hr-TB group (7 cases, 14.9%) was no significantly lower than that in the RR-TB group (4 cases, 11.8%) (*P* = 0.754).

**Conclusion:**

The treatment of retreated Hr-TB patients was difficult and could be statistically similar or considerably worse than RR-TB. It’s urgent to conduct further evaluation of the treatment status quo to guide the guideline development and clinical practice of Hr-TB patients.

## Introduction

Isoniazid (INH, H) has been widely used in the tuberculosis treatment as early as the early 1950s, and initially the anti-tuberculosis regimen was the traditional consisting of isoniazid, streptomycin and para-aminosalicylic acid for a course of 12–18 months. In the 1960s and 1970s, a new 6-month short-course chemotherapy regimen (2HREZ/4HR) was further developed, which has been used in China from the early 1980s to date [1]. Isoniazid is an important first line TB drug with commonly drug resistant mutation [2]. As we know, the development of drug resistance may be associated with several human factors, such as the continuous use of low-dose TB drugs at the time of initial treatment as a trigger for drug resistance [3], and the constant evolution of Mycobacterium tuberculosis (MTB) as a result of external factors, which in turn leads to MTB cell wall mutation [4]. With regard to the comparison of isoniazid and rifampicin application, the history of clinical application of isoniazid is longer than that of rifampicin, and the scope of isoniazid application for the treatment of tuberculosis is larger than that of rifampicin. The bactericidal strength of isoniazid and rifampicin is comparable, and the treatment failure rate of Hr-TB patients is higher than that of RR-TB patients; the incidence of isoniazid resistance is higher than that of rifampicin resistance, according to epidemiological investigation data in China [5]. However, Hr-TB has not received the same attention as rifampicin resistance; for example, rifampicin resistance may be considered as MDR-TB, and treatment regimens for isoniazid resistance have consistently been weaker than those for rifampicin resistance [6–10].

World Health Organization recommended treatment regimen for Hr-TB patients receiving rifampicin (RIF,R), ethambutol (EMB,E), pyrazinamide(PZA,Z) and levofloxacin (Lfx) for 6 months [8, 9], but without new concrete recommendations for retreated Hr-TB patients. Few studies have paid insufficient attention to the retreated Hr-TB and its’ treatment outcomes are no less than RR-TB does [11].

China is among the 30 high TB burden countries worldwide and only a few studies have investigated the clinical features and risk factors related to tuberculosis recurrence. Therefore, we compared the efficacy and safety data among Hr-TB and RR-TB in bacteriologically positive retreated pulmonary tuberculosis cases.

## Materials and methods

### Study design

**Fig. 1:**
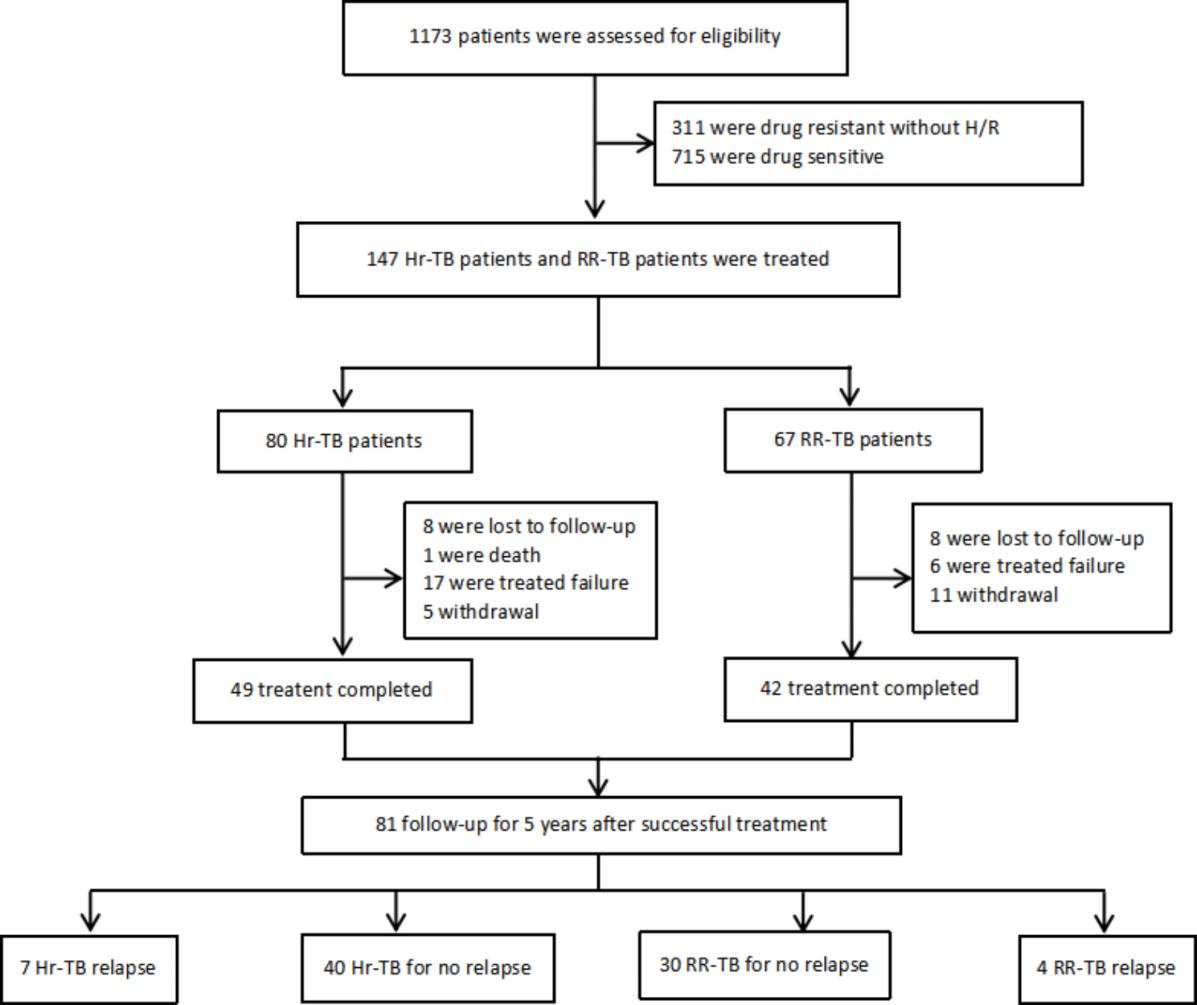
Flowchart of the study design

A retrospective study was used to collect bacteriologically positive retreated patients with isoniazid/rifampicin resistant pulmonary tuberculosis, who were conducted at 29 tuberculosis control institutions in China from July 2009 to June 2021 and followed up for 5 years after discontinuing the medication at the end of the full treatment course (Fig. 1), which is to compared the efficacy and safety data of outcomes among Hr-TB and RR-TB in bacteriologically positive retreated pulmonary tuberculosis cases from this study in this participant sub-group. This study was approved by the Ethics Committee of Beijing Chest Hospital affiliated with Capital Medical University (2009-12) and registered in the Chinese Clinical Trial Registry (*ChiCTR2100048701 and ChiCTR1800017441*). Written informed consent was obtained from each participant prior to enrollment.

The laboratory-confirmed Hr-TB/RR-TB patients received the individualized treatment regimen depended on the H/R single or multi drug resistance, consisting of at least 4–5 effective drugs selected from RIF (R), INH (H), ethambutol (EMB, E), pyrazinamide (PZA, Z), Para-aminosalicylic acid (PAS), rifampicin (L), amikacin (Am)/capreomycin (Cm), prothionamide (Pto) and Levofloxacin (Lfx)/moxifloxacin (Mfx). Am was used for 4–6 months in intensive phase and Lfx and PZA were used to replace H/R throughout the whole treatment course. Pto was added into the treatment regimen when application conditions were met for some patients. The new drugs such as linezolid, clofazimine, cycloserine, bedaquiline, and delamanid were not included in the treatment regimens, so as to reserve sufficient sensitive drugs for future retreatment once treatment failed or progressed to MDR-TB.

### Study patients

After excluding those who were with MDR/XDR-TB and incomplete data regarding drug sensitivity test, a total of 1173 participants were enrolled in the study at 29 sites and 147 patients were bacteriologically positive isoniazid/rifampicin resistant, of which 80 were isoniazid-resistant and 67 were rifampicin-resistant from July 2009 to June 2021. The inclusion criteria of cases are (1) no allergy to tuberculosis drugs, with normal liver and kidney function and routine blood and urine test results; no comorbid pneumoconiosis, HIV infection, hepatitis B, hepatitis C, cirrhosis, non-tuberculous mycobacteria lung disease, immune system diseases and severe cardiopulmonary insufficiency; (2) aged 18–65 years; (3) not pregnant; (4) sputum smear positive for acid-fast bacilli and culture positive for *M*. tuberculosis, *M*. tuberculosis complex for strain identification result, and availability of drug sensitivity test results before the current treatment; (5) agree to receive retreatment and sign the informed consent form; (6) complete record of patient information. Cases were excluded if patients with indicated sensitivity in drug sensitivity test for retreatment TB, and or patients with drug resistance other than isoniazid resistance or rifampicin resistance (e.g., those with ethambutol and/or streptomycin resistance were excluded), as well as patients with MDR, XDR and NTM lung disease. Patient demographics, comorbidities, medical history, bacteriologic information, radiologic data, detailed treatment information, adverse events, treatment outcomes, and post-treatment follow-up information were extracted through individual chart review from the electronic information system.

### Definitions

Definitions and treatment outcome were defined as the WHO guidelines [12].

Hr-TB is the tuberculosis caused by isoniazid-resistant, rifampin-susceptible Mycobacterium tuberculosis, including H mono-resistance and H poly-resistance.

RR-TB is the tuberculosis caused by rifampin-resistant Mycobacterium tuberculosis strains that may be isoniazid-susceptible or isoniazid-resistant or resistant to other first- or second-line TB drugs.

Cure was defined as cases in which the patient completed the treatment according to the program and provided two consecutive negative sputum smear results, including one at the end of the treatment.

Completed treatment was defined as cases in which the patient had completed the treatment according to the program protocol but did not meet the definition for cure because of lack of bacteriological results.

Additionally, the cured and completed treatment categories were combined as ‘‘treatment success,” whereas the other categories were combined as ‘‘poor treatment outcome’’.

Recurrence: disease recurrence after initial cure or treatment complete, without genotypic evidence of the same organism by 24-loci Mycobacterial Interspersed Repetitive Unit-Variable Number of Tandem Repeats (MIRU-UNTR) testing.

For the purpose of this study, a successful outcome included patients the definition of Cure or Treatment Completed while an unsuccessful outcome included patients the definition of Treatment Failure, Acquired Drug Resistance, Death or Relapse.

### Study assessments

All enrolled retreated patients with bacteriologically positive TB were managed at home, and each of them were required to receive sputum smear and culture examination, liver function, kidney function, routine blood and urine tests every month during the intensive phase and once every two months during the continuation phase. Chest X-rays were performed every three months during the treatment period. Patients were required to report at any time in the case of uncomfortable symptoms. All testing results were evaluated and reviewed once every month by the expert group.

### Quality control

The designated data administrator is responsible for data entry and management of electronic information system. Two trained staff were responsible for evaluating and checking all cases information. The national reference laboratory technicians provided laboratory testing training to the technicians of each partner to ensure uniform standards.

Personal information on cases was acquired from the case report form and electronic medical records. The case report form was filled by the researcher and each case must complete the case report form. The completed case report form is reviewed by the clinical inspector and the first page is handed over to the data manager for data entry and management.

### Statistical analysis

Statistical analysis were conducted by using SPSS 19.0. The categorical data was presented by number of cases (percentage), and the χ^2^ test or exact probability method was used for comparison between groups. Differences were considered statistically significant if *P* < 0.05.

## Results

### Patients’ characteristics

A total of 147 cases were included in analysis. 112 (76.1%) of 147 patients were male and 35 (23.8%) were female. 80 (54.4%) patients were Hr-TB patients and 67 (45.5%) were RR-TB patients. The differences in general information (age, sex, body mass, type of retreatment and comorbid diabetes) between the two groups were not statistically significant (*P* > 0.05). The patients’ characteristics are reported in Table 1.

**Table 1:** Demographic and clinical characteristics

Variables	Hr-TB		RR-TB		*χ* ^2^	*P*
	n	%	n	%		
Age group					0.276	0.871
18–39	34	42.5	30	44.8		
40–59	39	48.7	30	44.8		
60-	7	8.8	7	10.4		
Sex					0.137	0.711
Female	20	25.0	15	22.4		
Male	60	75.0	52	77.6		
BMI group					0.552	0.458
≥ 18.5	54	67.5	49	73.1		
< 18.5	26	32.5	18	26.9		
Type of retreatment					5.618	0.060
Relapse	45	56.3	49	73.2		
Initial treatment failure	12	15.0	9	13.4		
Other retreatment	23	28.7	9	13.4		
Comorbid diabetes					0.900	0.343
Yes	4	5.0	6	9.0		
No	76	95.0	61	91.0		

Note: “-” means that the Fisher exact probability method was used for the analysis

### Comparison of related treatment variables between Hr-TB and RR-TB group

There was statistically significant difference (χ^2^ = 6.077, *P* = 0.014) in the number of lesions involving lung fields at the beginning of the treatment between Hr-TB and RR-TB group, and the rate of number of lesions involving lung fields ≥ 3 in Hr-TB group 75.9% (60/79) was higher than RR-TB group 56.7% (38/67). There was no statistically significant difference (χ^2^ = 1.503, *P* = 0.472) in the number of lung cavities at the beginning of the treatment between Hr-TB and RR-TB group. The negative sputum smear rates at the end of the second month of treatment were 65.7% (46/70) and 85.7% (42/49) in the Hr-TB and RR-TB group, respectively, with statistically significant difference (χ^2^ = 4.992, *P* = 0.025). The negative sputum culture rates at the end of the second month were 61.2% (36/59) and 69.2% (27/39) in the two groups, respectively; there was no statistically significant difference (χ^2^ = 0.379, *P =* 0.538) in the negative sputum smear rates at the end of the sixth month between two groups, but the negative sputum smear rate in Hr-TB group 80.9% (51/63) was 10% lower than RR-TB group 90.5% (38/42); there was no statistically significant difference (χ^2^ = 1.109, *P =* 0.292) in the negative sputum culture rates at the end of the sixth month between two groups, but the negative sputum culture rate in Hr-TB group 76.4% (42/55) was nearly 20% lower than RR-TB group 91.7% (33/36); the incidences of serious adverse reactions were 2.7% (2/73) and 2.2% (1/45), respectively, and the difference was no statistically significant (*P* > 0.05). There was no statistically significant difference (*P =* 0.166) with regard to the treatment outcomes of the two groups, the cure rates were 54.7% (41/75) and 53.6% (30/56), respectively, and the failure rate in Hr- TB group 22.7% (17/75) was 10% higher than RR-TB group 10.7% (6/56). There was no statistically significant difference (*P =* 0.164) in chest x-ray change between two groups and the rate of unchanged chest x-ray in Hr- TB group 18.7% (14/76) was 11% higher than RR-TB group 7.4% (4/54). See Table 2.

**Table 2:** Comparison of related treatment variables for patients with Hr-TB or RR-TB

Variables	H-resistant		R-resistant		*χ* ^2^	*P*
	n	%	n	%		
number of lesions involving lung fields					6.077	0.014
≤ 2	19	24.1	29	43.3		
≥ 3	60	75.9	38	56.7		
number of lung cavities					1.503	0.472
0	31	38.8	33	48.5		
1–2	39	48.7	27	39.7		
≥ 3	10	12.5	8	11.8		
Sputum smear at the end of month 2					4.992	0.025
Negative	46	65.7	42	85.7		
Positive	24	34.3	7	14.3		
Sputum culture at the end of month 2					0.379	0.538
Negative	36	61.0	27	69.2		
Positive	23	39.0	12	30.8		
Sputum smear at the end of month 6					1.109	0.292
Negative	51	80.9	38	90.5		
Positive	12	19.1	4	9.5		
Sputum culture at the end of month 6					2.54	0.111
Negative	42	76.4	33	91.7		
Positive	13	23.6	3	8.3		
Treatment outcome					-	0.166
Cure	41	54.7	30	53.6		
Treatment completed	8	10.7	12	21.4		
Failure	17	22.7	6	10.7		
Death	1	1.3	0	0.0		
Lost	8	10.6	8	14.3		
Serious adverse reaction					-	1.000
Yes	2	2.7	1	2.2		
No	71	97.3	44	97.8		
Chest x-ray change					-	0.164
Abated	59	78.7	48	88.9		
Unchanged	14	18.7	4	7.1		
Deteriorated	2	2.6	2	4.0		

Note: “-” means that the Fisher exact probability method was used for the analysis

### Follow-up

**Fig. 2:**
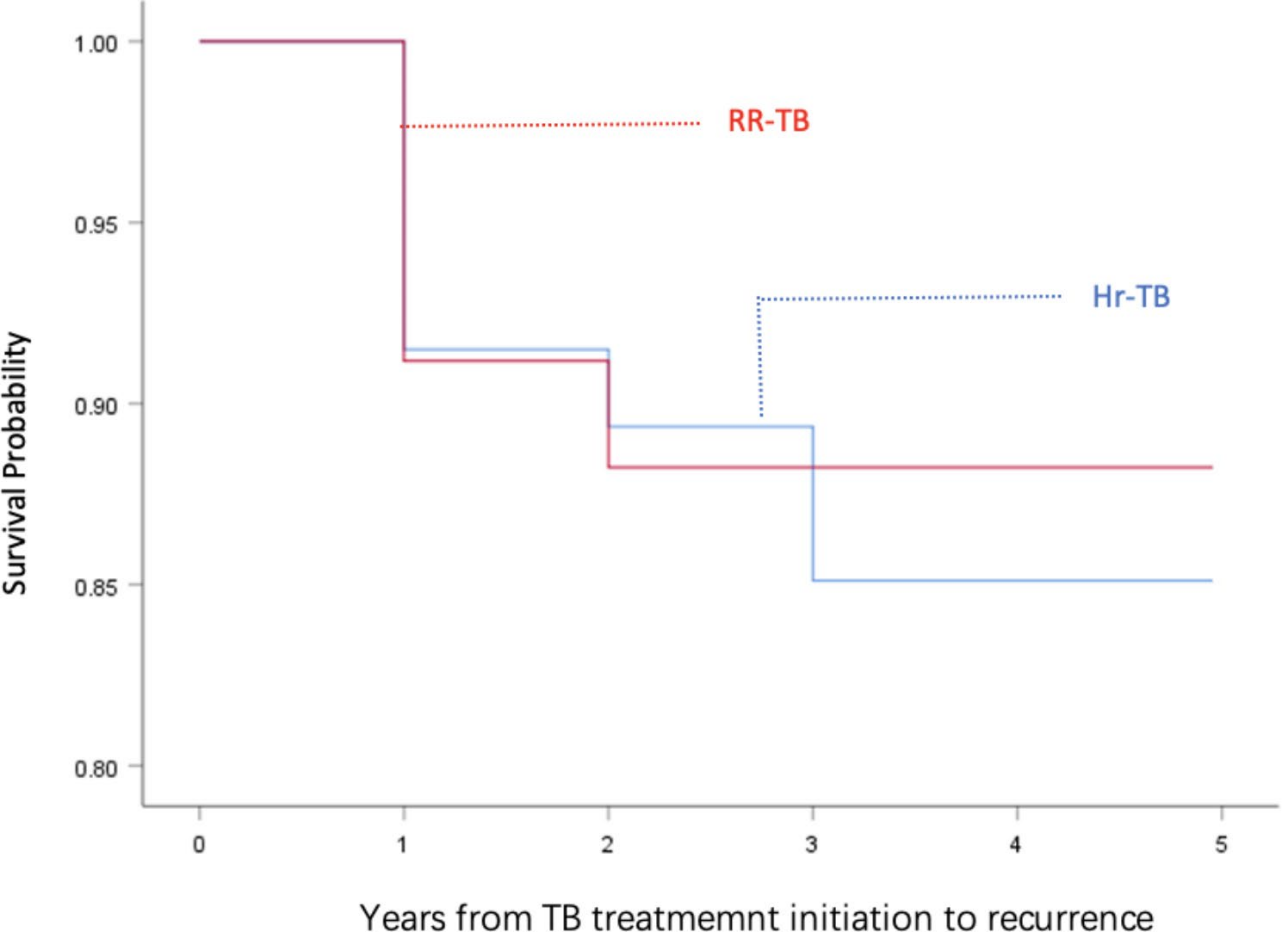
Time to TB recurrence among successfully treated patients, stratified by Hr-TB group and RR-TB group

Total 81 participants were completed the 5-year follow after the successful treatment and there were 11 cases of recurrence. Difference of time to recurrence in relation to Hr-TB and RR-TB group was not significant (*P* = 0.754). 47 cases were successfully treated in Hr-TB group with a relapse rate of 14.9% (7/47), and 34 cases were successfully treated in RR-TB group with a relapse rate of 11.8% (4/34)). See Fig. 2.

## Discussion

Retreated Hr-TB has not received much attention until recent years [8, 9, 13, 14]. In fact, clinical treatment of Hr-TB cases in the early years was also highly challenging, but there were few data to confirm it. This can be confirmed and validated by the data accumulated by this study over approximately 10 years. In this study, for example, the sputum smear negative rate at the end of the second month in the Hr-TB group was lower than that in the RR-TB group (*P* < 0.05), the negative sputum smear rate in Hr-TB group 80.9% (51/63) was 10% lower than RR-TB group 90.5% (38/42), the negative sputum culture rate in Hr-TB group 76.4% (42/55) was nearly 20% lower than RR-TB group 91.7% (33/36), the failure rate in Hr- TB group 22.7% (17/75) was 10% higher than RR-TB group 10.7% (6/56), and the rate of unchanged chest x-ray in Hr-TB group 18.7% (14/76) was 11% higher than RR-TB group 7.4% (4/54). The results showed that Hr-TB patients had the unfavorable outcomes compare to the RR-TB patients. Besides, the prevalence of Hr-TB patients was higher than the prevalence of rifampicin resistance globally and many patients with Hr-TB were missed by current diagnostic algorithms driven by rifampicin testing [15]. Hr-TB patients therefore should be paid more attention and further researches are needed just as RR-TB patients.

With regard to the treatment outcome of patients in this study, despite the individualized treatment, the treatment success rate of Hr-TB cases is still lower than that of RR-TB cases, and the treatment efficacy is still unsatisfactory. First, this is related to the insufficient attention to the chemotherapy regimen for Hr-TB cases. In addition, treatment failure is closely associated with treatment regimen, low dose of Lfx (0.4/d-0.5/d) used by Chinese physicians according to the drug instructions, the limited individualized treatment skills of some physicians, and the failure to timely change the treatment regimen for drug-resistant patients [16]. Besides, the unfavorable treatment results in Hr-TB group may be related to the serious lesions involving lung fields at the beginning of the treatment, but it was need to confirm through further study. The WHO mentioned high-dose H in the treatment of MDR-TB [6], and the results of this study showed that the clinicians should raise awareness on the dosage of H medication for patients who have undergone retreatment, have treatment failure or relapse with conventional dosage of H, or sensitive to H.

According to the analysis of successfully treated patients during the 5-year follow-up period after medication discontinuation, it was found that the relapse rates in the Hr-TB and RR-TB group were not statistically significant. However, the relapse rate in the Hr-TB group (14.9% (7/47)) was higher than that in the RR-TB group (11.8% (4/34)), which may be related to the smaller sample size in the Hr-TB or RR-TB groups, or to the fact that the H-resistance regimen was not as robust as the R-resistance regimen. It is suggested to carry out the relevant studies to further verify the recurrence of Hr-TB and RR-TB patients.

Some studies have confirmed that the treatment regimen for treated Hr-TB patients was weak when only use Lfx to replace H, because it’s cannot achieve sterilization when the dose of Lfx was not enough [6–9, 13]. Therefore, only H replacement resulted in a poor treatment outcome for retreated pulmonary Hr-TB patients before. According to the study, although there were no statistical significance differences in the sputum negative conversion rate at the end of six months between Hr-TB and RR-TB group, the sputum negative conversion rate of Hr-TB group was obviously lower than RR-TB group, as well as the treatment failure rate and 5 years follow up recurrence rate were obviously higher than RR-TB group. The RR-TB patients has been getting broad attention compare to Hr-tb patients, which was also proposed to be regarded as the MDR-TB patients [17]. It can be seen that the importance of Hr-TB was less than that of RR-TB, resulting in the difference between this two groups.

This study suggested that the resistance of an effective bactericide drug such as H, its drug resistance cannot be simply replaced by an antibacterial drug. The design of treatment regimen for the retreated pulmonary TB patients with the resistance of an effective bactericide drug such as H need to consider its treatment history and susceptibility results of drugs, instead of replacing one drug. For retreated pulmonary TB patients, particularly in patients who have been used all first line drugs or first treatment failure (second treatment), their drug sensitivity may be reduced, but does not exceed the limit of drug resistance, so that the reliability and reference of susceptibility results or the uncertainty close to the critical value of drug resistance should be taken into consider when selected a new regimen [18]. It is suggested that the management of retreated Hr-TB patient should also be regarded as MDR-TB patients.

The difference between Hr-TB and RR-TB patients in this study was statistically significant only in the negative sputum smear rate at the end of the second month of treatment, while the differences in sputum culture at the end of the second and six month were not statistically significant, which may be related to the fact that the patients excreted dead bacteria during treatment course and the sputum smear test had low sensitivity and could not distinguish between live and dead bacteria; this suggests that the negative sputum smear rate at the end of the second month of treatment may not be of practical significance, but at least it could indicate that the treatment outcomes of Hr-TB and RR-TB patients are comparable.

Our study has some limitations. The sample size of isoniazid and rifampicin resistant groups may be statistical biases due to the lack of detailed stratification. Besides, due to the lack of sputum in the late stage of treatment or treatment failure, death, loss, etc. for some patients, it’s unable to analyze the sputum bacteria results at the end of the treatment.

In conclusion, it is suggested that retreated patients with isoniazid resistant pulmonary tuberculosis should be considered as MDR management as well as RR-TB patients. This is an active response to the management of Hr-TB patients so as to prevent the development and spread of MDR-TB.

## Data Availability

All data generated or analyzed during this study are included in this published article. The data that support the findings of this study are available from the corresponding author upon reasonable request.
